# Oral immunotherapy for cow’s milk allergy in children: a systematic review and meta-analysis

**DOI:** 10.3389/fimmu.2025.1570050

**Published:** 2025-06-04

**Authors:** Yan Wang, Shunli Liu, Meizhu Lu, Jingyu Guo, Can Lv, Lan Huang

**Affiliations:** ^1^ Department of Emergency, West China Second University Hospital, Sichuan University, Chengdu, China; ^2^ Key Laboratory of Birth Defects and Related Diseases of Women and Children (Sichuan University), Ministry of Education, Chengdu, China; ^3^ Department of Pediatrics, West China Second University Hospital, Sichuan University, Chengdu, China

**Keywords:** oral immunotherapy, cow’s milk allergy, children, desensitization, adverse reaction

## Abstract

**Objective:**

Cow’s milk allergy (CMA) is one of the most common causes of food allergies (FA) in children. There have been studies on the use of immunotherapy in cow’s milk protein allergy, with oral immunotherapy (OIT) being the most extensively researched. We conducted a comprehensive analysis of randomized controlled trials (RCTs) to explore the efficacy and safety of OIT to manage cow’s milk allergy in children.

**Methods:**

PubMed, EMBASE, Cochrane Library, and Scopus databases were searched from their inception until August 2024. Randomized controlled trials that reported on the efficacy or safety of IT for CMA were included. Two investigators independently extracted data on regimen of intervention, outcomes, number of cases and gender ratio. Pooled estimates of relative risks or standardized mean differences with 95% confidence intervals were calculated from the included studies for dichotomous and continuous outcomes.

**Results:**

Nineteen RCT articles (815 participants) were included. The meta-analysis indicated that oral immunotherapy significantly facilitated desensitization in patients with cow’s milk allergy in children (relative risk [RR] 2.51, 95% CI: 1.54–4.09, I²=84.4%). Tolerance threshold at oral food challenges (OFC) increased following oral immunotherapy compared with a standard mean difference (SMD) of 3.58 (2.82–4.33). After oral immunotherapy, the antibody titers of cow milk protein sIgE (SMD -0.42, 95% CI: -0.72 to -0.11, I²=28.8%) and casein sIgE (SMD -0.54, 95% CI: -0.97 to -0.11, I²=0%) decreased. The risk of adverse reactions with immunotherapy was not higher than that in the control group, with an RR of 2.05 (95% CI 0.96–4.37, I²=81.5%).

**Conclusions:**

Oral immunotherapy, is associated with desensitization to CMA in children, without increased risk of short-term adverse events, but late complications such as eosinophilic esophagitis require caution. More high-quality studies are needed to explore the long-term efficacy of OIT for CMA.

**Systematic review registration:**

https://www.crd.york.ac.uk/PROSPERO/recorddashboard, identifier CRD42024541769.

## 1 Introduction

Food allergy (FA) as a common disease in children with increasing prevalence rates over the past two to three decades. In some countries, FA prevalence has reached as high as 10% (1). A systematic review estimated that direct medical costs for patients with FA in the United States alone amount to $4.3 billion annually (2). Cow’s milk allergy is one of the most common causes of FAs in children. The reported prevalence of CMA varies due to differences in diagnostic methods and epidemiological survey designs. CMA prevalence range of 1.8%–7.5% in the first year of life was reported (3). Some adult patients with CMA had allergies since childhood, but some studies have also reported adult-onset CMA (4).

Management of cow milk protein allergy involves strict avoidance of food allergens and the use of pharmacological treatments to address reactions from accidental exposure. In infancy, CMA is often managed by substituting cow milk with extensively hydrolyzed protein or amino acid-based formulas. However, these alternatives have poor taste and are costly, imposing a significant financial burden on families (4). Alongside the persistent risk of systemic allergic reactions, avoiding milk consumption may also have nutritional implications. Sinai et al. compared the adult height of CMA patients with a control group without dietary restrictions and found that CMA patients were, on average, 3.8 cm shorter and had a higher rate of vitamin D deficiency (5). Recently, there has been an increasing research focus on immunotherapy for CMA, aiming to achieve desensitization as early as possible (6). Various immunotherapy approaches for milk protein desensitization, including oral immunotherapy, sublingual immunotherapy, and subcutaneous immunotherapies, and OIT is the most common approach. OIT is a treatment method that induces immune tolerance by gradually increasing the intake of allergens. OIT likely induces immune tolerance via repeated allergen exposure by rebalancing the Th2-driven allergic response toward a Th1/Treg-dominated profile (7). However, findings regarding their effectiveness are inconsistent. Some studies have found that immunotherapy can facilitate desensitization to cow milk protein (8), while others have reported no such effects (9). Therefore, we aimed to conduct a systematic review and meta-analysis of oral immunotherapy for CMA in children to provide clinical recommendations for managing CMA.

## 2 Materials and methods

This review follows the Preferred Reporting Items for Systematic Reviews and Meta-Analyses (PRISMA) guidelines (10). The protocol and search strategies were registered with PROSPERO (York. ac. uk) (CRD42024541769).

### 2.1 Retrieval of studies

A systematic search of PubMed, EMBASE, the Cochrane Library, and Scopus was conducted to identify relevant studies from inception to August 2024. The search strategy is detailed in **Supplementary Material**. Additional studies were identified by manually searching the references of included research articles, meta-analyses, and reviews.

### 2.2 Eligibility criteria

The eligibility criteria were as follows: (1) participants were under 18 years of age with a confirmed IgE-mediated milk protein allergy; (2) the intervention involved IT; (3) studies reported on at least one of the following outcomes: efficacy (desensitization or sustained unresponsiveness [SU]), changes in reaction thresholds during oral food challenges (OFC), laboratory parameters, changes in quality of life, or adverse reactions (ARs); and (4) studies had a RCT design.

Exclusion criteria were: (1) non-IgE-mediated milk protein allergy; (2) absence of a control group; (3) case-control studies, case reports, conferences, reviews, and animal studies; (4) overlapping data or lack of raw data; and (5) participants over 18 years of age.

### 2.3 Data extraction

Two independent reviewers extracted data on the first author, publication year, country, study design, intervention, comparator, sex ratio, number of cases and total sample population, OIT regimen, and outcomes.

### 2.4 Quality assessment

The Cochrane risk-of-bias tool was used to assess the quality of randomized trials. RoB grades bias of random sequence generation, allocation concealment, blinding of participants and personnel, blinding of outcome assessment, incomplete outcome data, selective reporting, and other biases of low, unclear, or high risk.

### 2.5 Statistical analysis

The meta-analysis was conducted using Stata version 18.0 (Stata Corporation, College Station, TX, USA). For trials with multiple reports, we included all relevant data. A random-effects model was employed as anticipated. Pooled estimates of relative risks (RRs) with 95% confidence intervals (CIs) were calculated for dichotomous outcomes. We combined continuous outcomes across studies using standardized mean differences. The heterogeneity between studies was assessed using the I^2^ statistic (significance level >50%). We also performed sensitivity analysis by sequentially excluding each study. Publication bias was visually assessed using Egger’s and Begg’s tests (significance level: P<0.05). Review manager 5.4.1 was used to evaluate the certainty of the evidence.

## 3 Results

### 3.1 Search results and study characteristics

**Figure 1:**
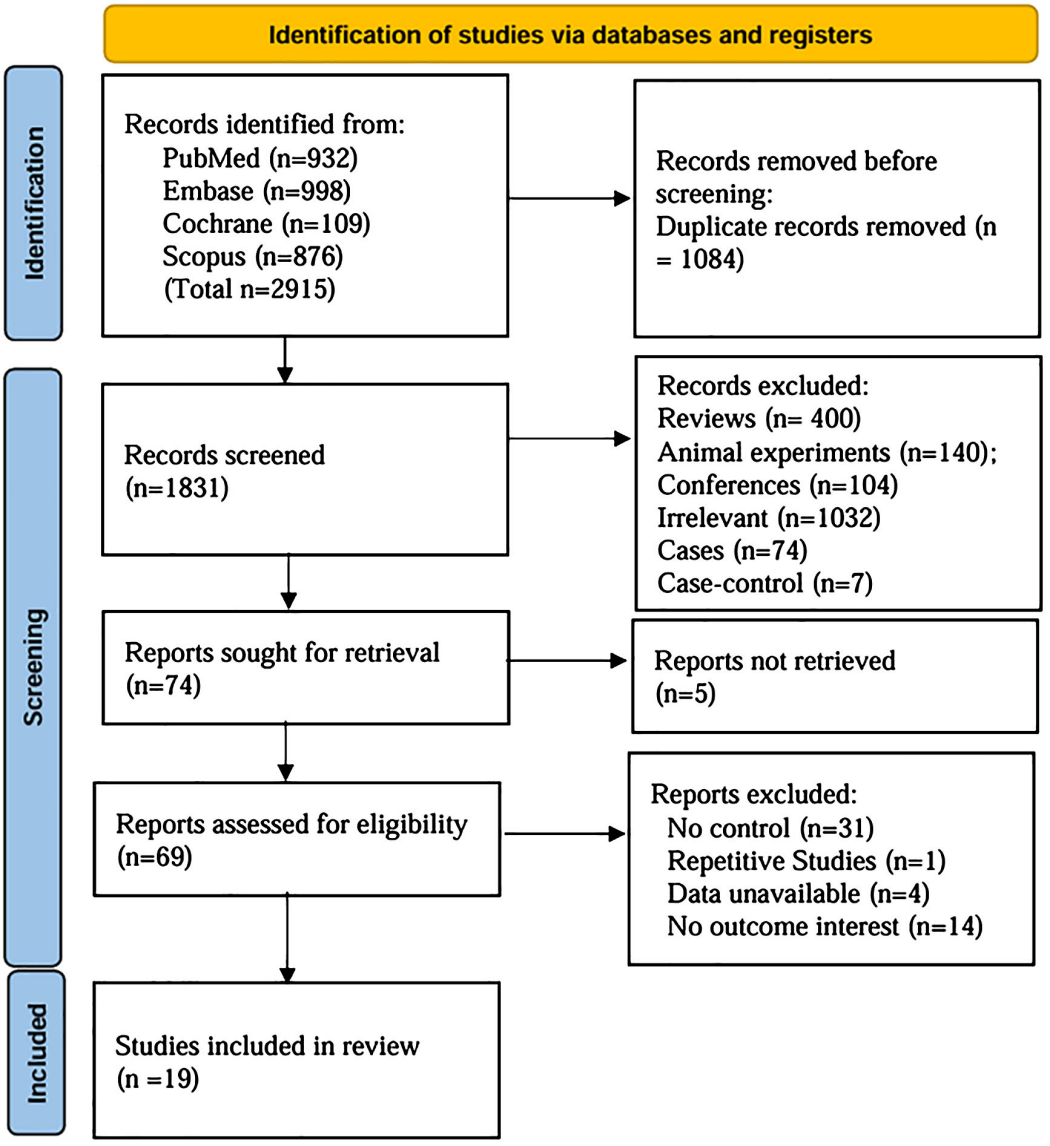
Flow diagram of the study selection process.

**Figure 2:**
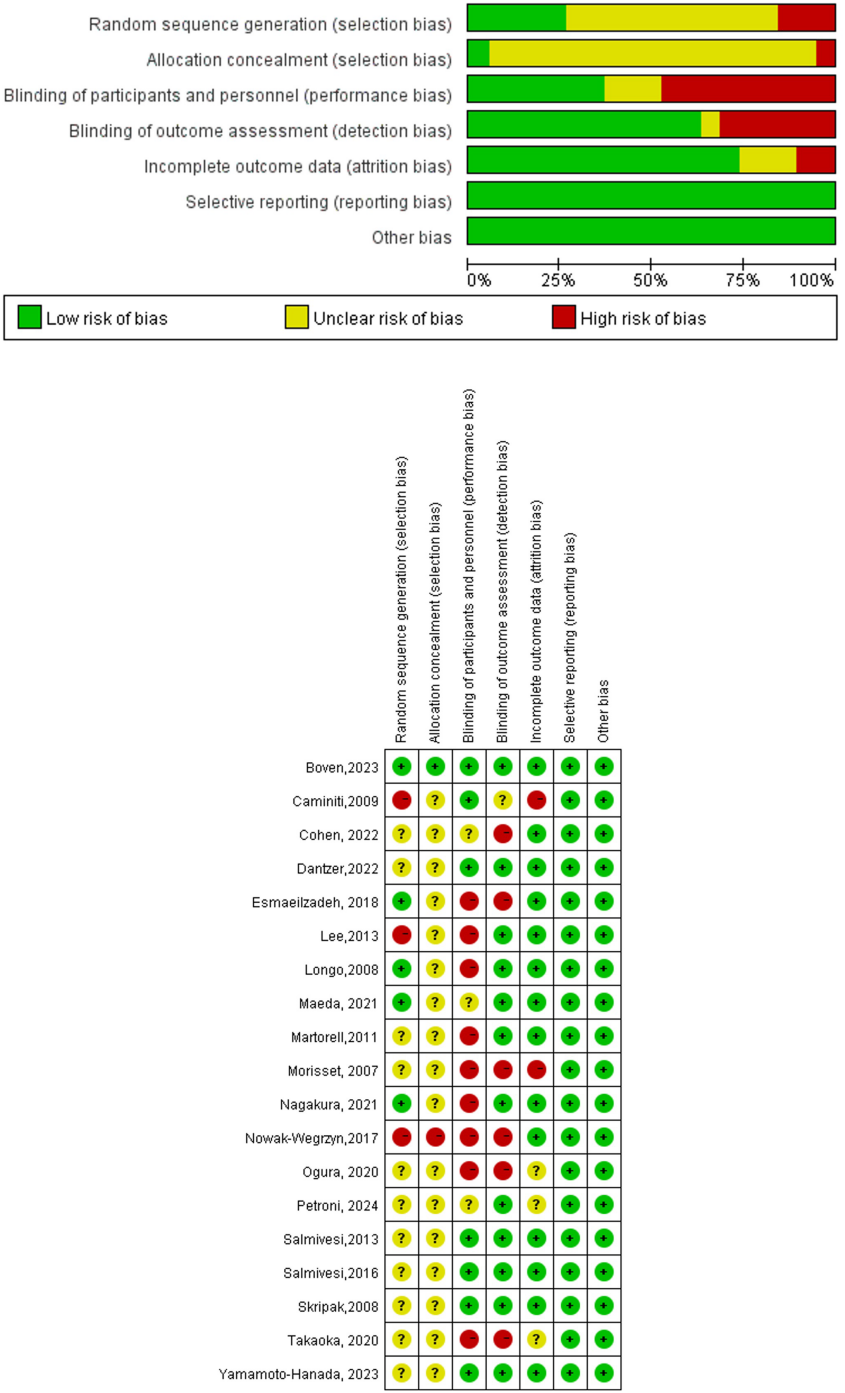
Risk of bias graph for studies on oral immunotherapy for IgE- mediated CMA in children.

A comprehensive search identified 2915 articles (PubMed: 932; EMBASE: 998; Cochrane Library: 109; Scopus: 876). After careful screening, 19 studies (8, 9, 11–27) were selected for inclusion (**Figure 1**). The characteristics of the 19 selected studies are presented in **Supplementary Table S1**. A total of 815 participants were enrolled, with 367 assigned to the control group and 448 to the OIT group. The ages of the study population ranged from 3 month to 18 years. Geographically, the studies were conducted in North America (5) (9, 13, 19, 23, 24), Europe (7) (8, 11, 20, 22, 25–27), and Asia (7) (12, 14–18, 21). Overall, the quality of the RCTs was low, with many studies showing unclear or high risk of bias in various methodological domains. The quality of evidence in the study was primarily influenced by allocation concealment and blinding procedures (**Figure 2**).

### 3.2 Primary outcomes

#### 3.2.1 Desensitization

**Figure 3:**
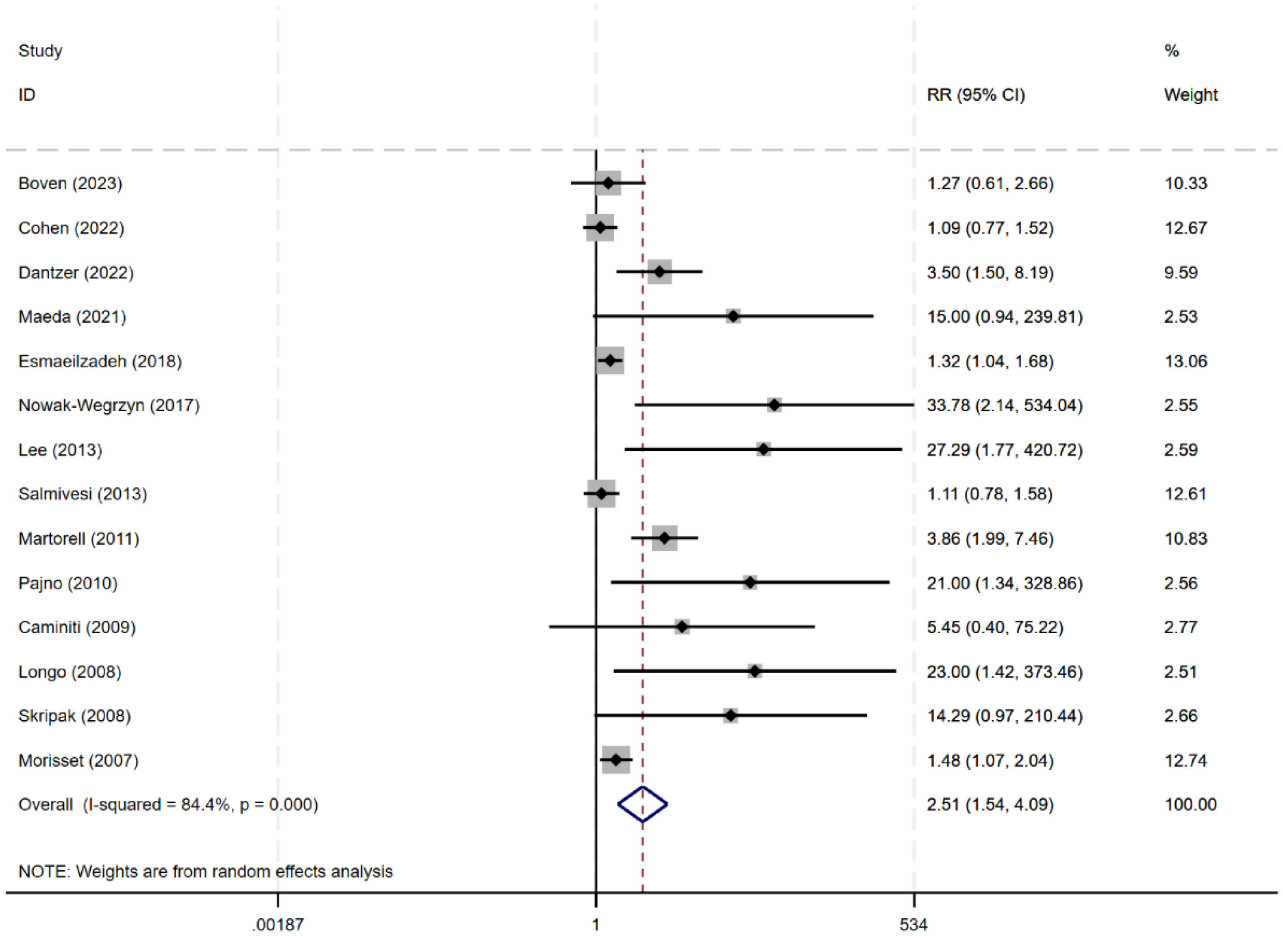
Metanalysis of desensitization induced by OIT in CMA.

Fourteen studies (n=663) (8, 9, 11, 13, 14, 18–26) evaluated the efficacy of desensitization across a wide range of thresholds (264 mg to 7250 mg, or 3–250 ml). The meta-analysis showed a significant increase in desensitization among patients allergic to milk (RR 2.51, 95% CI: 1.54–4.09), with an I² of 84.4%, indicating high heterogeneity (**Figure 3**). Further subgroup analyses were performed based age group, control group type, type of milk intervention, and treatment duration.

For children under 4 years of age, oral immunotherapy did not demonstrate effectiveness in the desensitization to cow’s milk protein (RR: 2.34, 95% CI: 0.91–6.06). However, for children over 4 years old, OIT was effective in inducing desensitization to cow’s milk protein (RR: 4.43, 95% CI: 1.41–13.94). This may be related to the fact that cow’s milk protein allergy in younger children has a certain degree of self-limiting nature, whereas the likelihood of spontaneous resolution of cow’s milk protein allergy in older children is lower. Compared to avoidance of cow’s milk protein intake (RR: 2.23, 95% CI: 1.23-4.05) or using soy milk as a control (RR: 10.37, 95% CI: 1.55-69.23), OIT achieved desensitization effects; but compared to a placebo control, OIT did not show effectiveness (RR: 4.55, 95% CI: 0.69-29.76). The combined data showed a statistically significant tolerance to cow’s milk in the OIT group using raw milk compared to the control group (RR: 3.00, 95% CI: 1.53-5.91, I²=84.9%), but using baked milk (RR: 3.80, 95% CI: 0.61-23.61) or heated milk (RR: 1.27, 95% CI: 0.61-2.66) as intervention methods did not show an advantage in desensitization (**Table 1**).

**Table 1:** Subgroup analysis of immunotherapy for cow’s milk allergy.

Variable		Included studies	Risk ratio* (95% CI)	I^2^
Means of intervention	Raw milk (8, 9, 14, 20–26)	10	3.00 (1.53-5.01)	84.9%
	Baked or heated milk (13, 18, 19)	3	3.80 (0.61-23.61)	91.9%
	Heated and glycated CM protein powder (11)	1	1.27 (0.61-2.66)	/
Control	Avoidance (8, 9, 14, 18, 19, 21, 26)	7	2.24 (1.23-4.05)	86.2%
	Placebo (13, 20, 24, 25)	4	4.55 (0.69-29.76)	91.2%
	Soy milk (22, 23)	2	10.37 (1.55-69.23)	0%
	Extensively hydrolyzed formula (11)	1	1.27 (0.61-2.66)	/
Intervention age	<4y (8, 11, 18, 21)	4	2.34 (0.91-6.06)	86.5%
	≥4y (9, 15, 20, 22–25)	7	4.43 (1.41-13.94)	89.3%
Treatment duration	≤24w (20–26)	7	4.46 (1.49-13.32)	88.4%
	>24w (8, 9, 11, 13, 14, 18, 19)	7	2.21 (1.21-4.06)	84.4%

#### 3.2.2 Change in reaction thresholds at OFC

**Figure 4:**
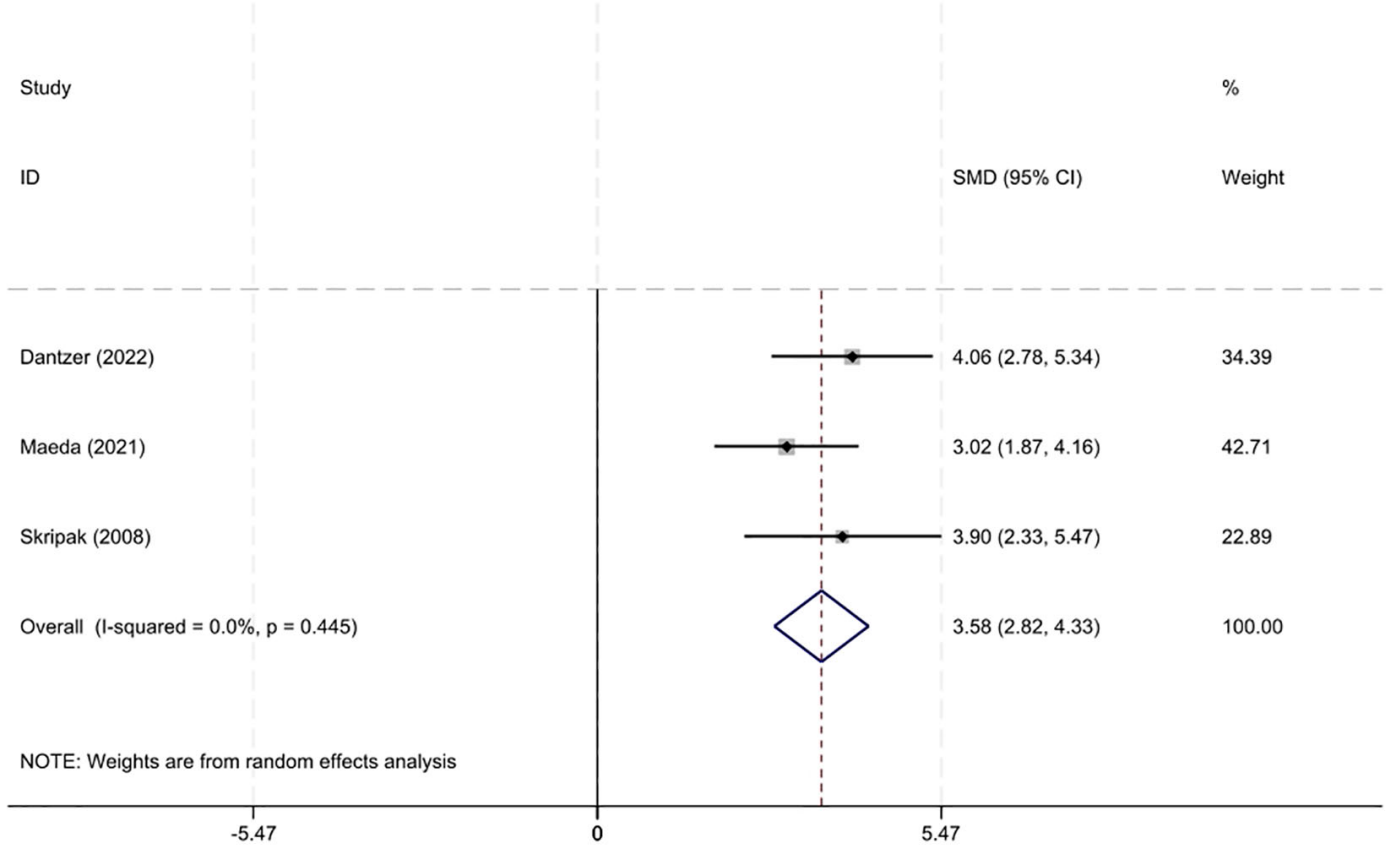
Metanalysis of change in reaction thresholds at OFC induced by OIT in CMA.

A meta-analysis (n=3) (13, 14, 24) indicated that the tolerance threshold at OFC increased following immunotherapy compared to before treatment, with a SMD of 3.58 (2.82–4.33) and an I² of 0% (**Figure 4**).

#### 3.2.3 Adverse reactions

**Figure 5:**
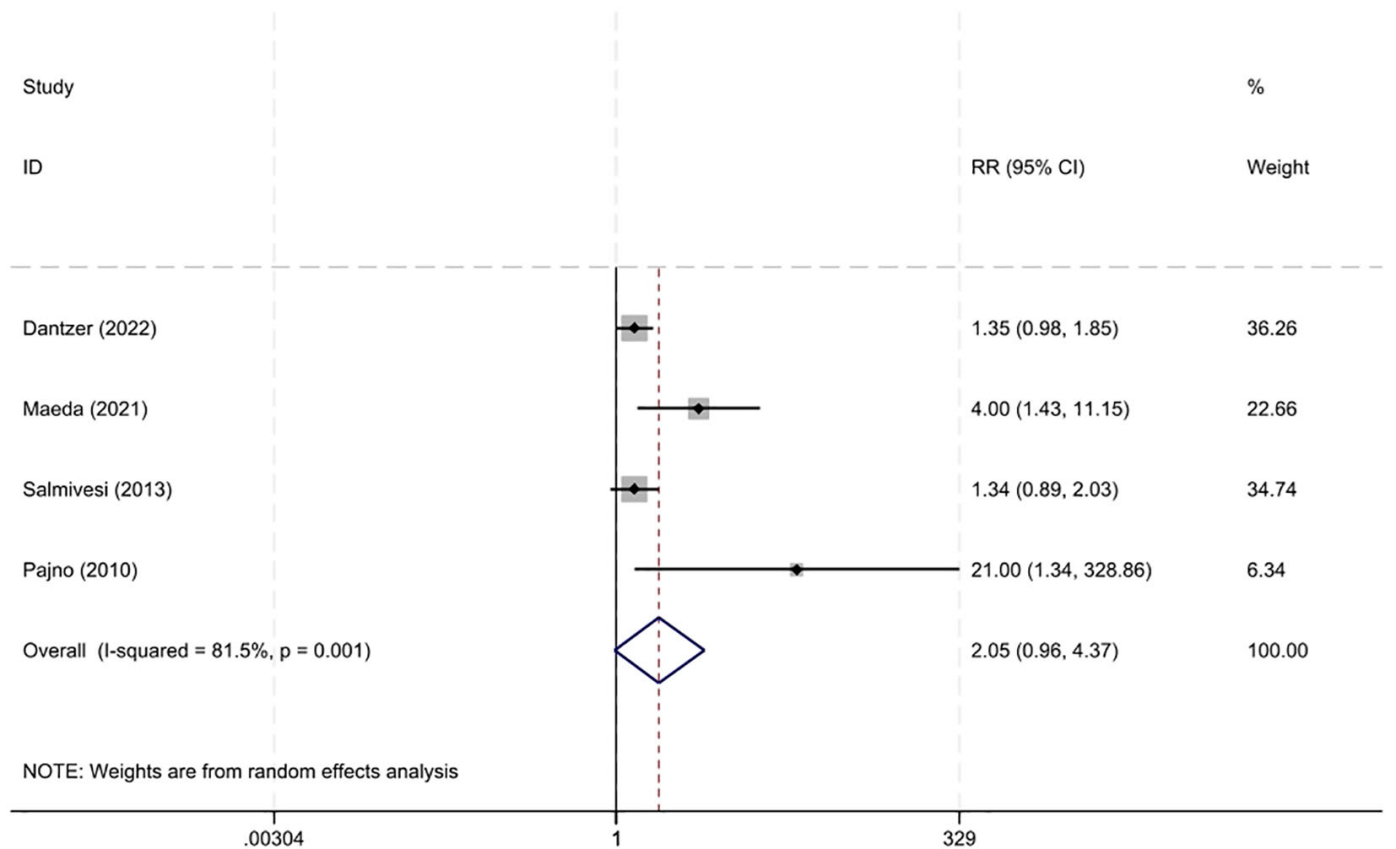
Adverse reactions of OIT in CMA.

Four studies (13, 14, 20, 22) documented the occurrence of adverse events. The risk of adverse reactions with immunotherapy was not higher than that in the control group, with an RR of 2.05 (95% CI 0.96–4.37, I²=81.5%) (**Figure 5**). Most adverse reactions were mild, and there was no significant difference in the risk of severe adverse reactions between the two groups, with an RR of 2.65 (95% CI 0.79–16.90, I²=0%) (13, 14, 22).

Eosinophilic esophagitis (EoE) is an allergen-mediated inflammatory disorder of the esophagus characterized by progressive esophageal dysfunction. EoE has been observed in patients receiving OIT for IgE-mediated food allergies and may represent a late complication of OIT treatment. Among the studies included in this meta-analysis, only one (15) reported a single case of EoE. After expanding our database search, we included 11 cohort studies (15, 28–37) and found that 4.0% (95% CI:2%-7%) of children receiving OIT had biopsy-confirmed EoE (**Supplementary Figure S1**).

### 3.3 Secondary outcomes

#### 3.3.1 Immunological changes

**Figure 6:**
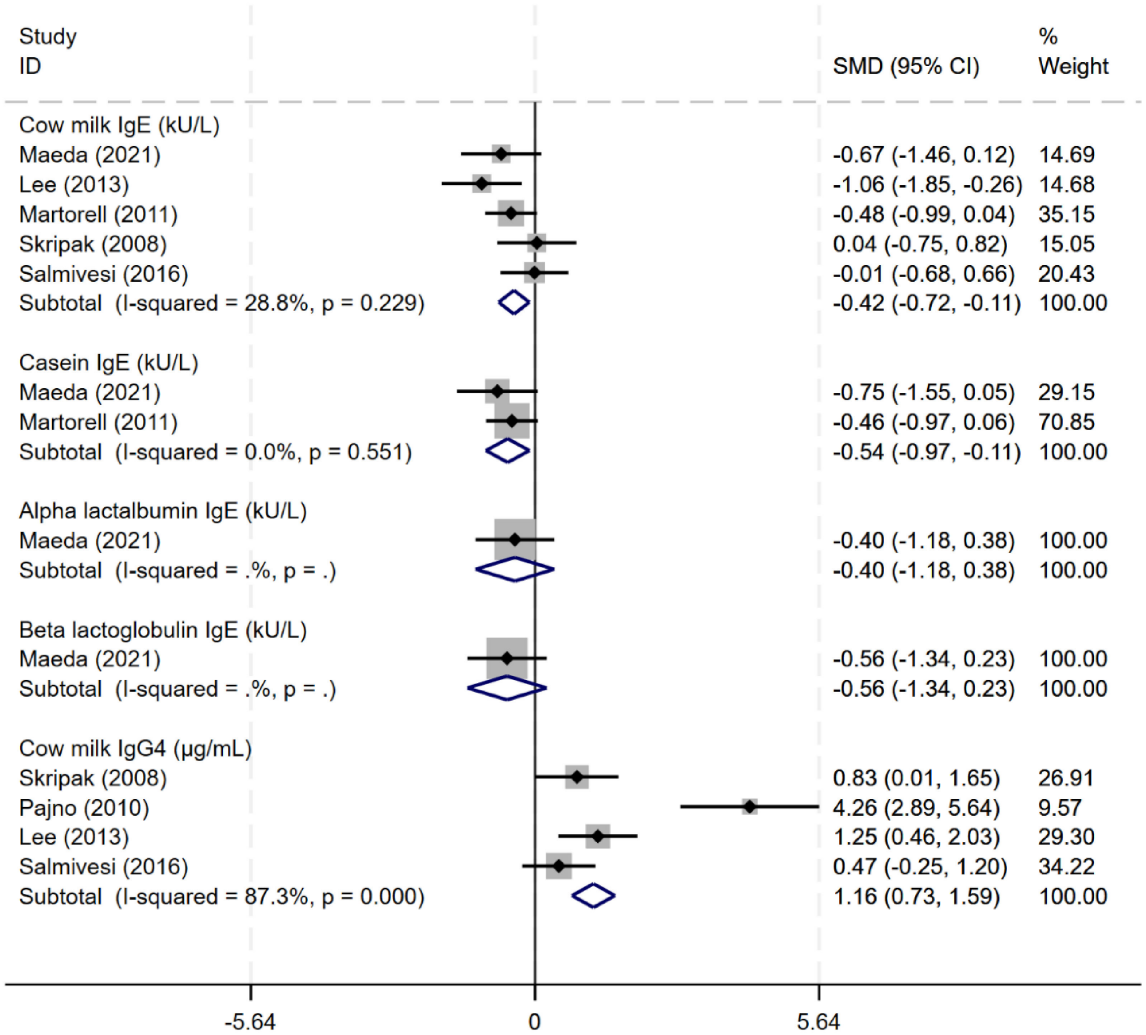
Metanalysis of immunological changes induced by OIT in CMA.

Five studies (8, 14, 21, 22, 24, 27) evaluated the effects of immunotherapy on immunological changes. Oral immunotherapy reduced the levels of serum cow milk protein-related (CM-sIgE) and casein-specific IgE (CN-sIgE) antibodies (CM-sIgE: SMD -0.42, 95% CI: -0.72 to -0.11, I²=28.8%; CN-sIgE: SMD -0.54, 95% CI: -0.97 to -0.11, I²=0%). After immunotherapy, cow milk protein IgG4 levels were higher than before treatment, with an SMD of 2.01 (95% CI 0.34 to 3.69, I²=89.1%). Only one study evaluated the change of α-lactalbumin and β-lactoglobulin IgE levels (**Figure 6**).

Only one study (27) in the literature reported the effects of OIT on cytokines. The analysis demonstrated that OIT did not significantly alter the levels of cytokines IL-4, IL-5, IL-6, or IL-10 (**Supplementary Figure S2**).

#### 3.3.2 Quality of life assessment

Only one study used a standardized scale to evaluate the impact of immunotherapy on the quality of life of patients (13), using improved Food Allergy Quality of Life-Parent Form (FAQOL-PF) or Food Allergy Quality of Life-Child Form (FAQOL-CF) scores (≥0.5-minimal clinically important difference) from baseline to month 12 as indicators. The RR was 0.58 (95% CI 0.18–1.85) for FAQOL-PF and 1.40 (95% CI 0.23–8.44) for FAQOL-CF.

#### 3.3.3 Other studies

Two studies (16, 17) compared the impact of different target doses on the efficacy of desensitization. No significant differences were observed between the low-dose (20–25%) and high-dose (100%) groups in the treatment of cow’s milk protein desensitization (RR 1.10, 95% CI 0.56–2.16). One study investigated the effect of adding probiotics to oral immunotherapy. The study found that the effect of OIT combined with the probiotic LP013216 on cow’s milk protein desensitization was not significantly different from that of OIT alone (RR 1.17, 95% CI 0.83–1.65). Only one study (15) compared the effects of heated milk versus raw milk on desensitization, showing no significant difference (RR 1.26, 95% CI 0.92-1.71).

### 3.4 Sensitivity analysis and publication bias

Sensitivity analysis was performed for the main outcomes with high heterogeneity. The results of sensitivity analysis confirmed stable results in the efficacy of OIT for CMA and adverse events of OIT (**Supplementary Figures S3**, **S4**). A funnel plot suggested a potential publication bias in relation to desensitization of OIT for CMA (Egger, P<0.001). We further undertook a sensitivity analysis by using the trim-and-fill method to estimate the number of missing studies that may have caused funnel plot asymmetry, and we imputed the hypothetical studies to produce a symmetrical funnel plot. After adjusting for potential publication bias, the impact of the intervention remained statistically significant (RR1.59, 95% CI 1.08-2.36) (**Supplementary Figure S5**).

## 4 Discussion

Our meta-analysis included 19 studies to comprehensively evaluate the overall efficacy and safety of immunotherapy in patients with IgE-mediated cow milk protein allergy. Despite the high heterogeneity and generally low evidence levels among these studies, immunotherapy was more effective for achieving desensitization in patients with CMA compared to avoidance therapy, and it significantly altered the cumulative tolerance dose. The immunotherapy group did not show a higher risk of adverse effects than the control group, and severe adverse events were rare.

Like the Riggioni (38) study, oral immunotherapy was effective in the treatment of cow’s milk allergy compared to avoidance of milk protein, with the results exhibiting considerable heterogeneity. Unlike the Riggioni study, which included eight RCT studies and two case-control studies and did not perform subgroup analysis, our study exclusively included RCTs, yet it still exhibited a high degree of heterogeneity, suggesting that the experimental design is not the source of heterogeneity. The differences may be related to the high heterogeneity in study populations, intervention protocols, outcome measures, follow-up periods, and other methodological variations. Despite the heterogeneity, sensitivity analysis suggested the reliability of the results. Standardization of trial design, outcome measurement, and reporting practices is crucial to address these issues and enhance the reliability and applicability of research findings.

CMA pathogenesis can be mediated by IgE, non-IgE, or both. Most cow milk protein allergies are mediated by IgE. Cow milk contains caseins and whey proteins, which account for approximately 80% and 20% of the total milk protein, respectively. Most patients with cow milk protein allergy are allergic to multiple proteins in cow milk, with casein, β-lactoglobulin, and α-lactalbumin being the major allergens (39). Immunological evaluation in this meta-analysis showed that immunotherapy reduced cow milk protein-specific IgE and casein-specific IgE levels. Levels of cow milk-related IgG4 increased, likely due to enhanced IgG4 binding to milk epitopes, which in turn reduces IgE binding to these epitopes and induces tolerance (40).

This meta-analysis also examined how intervention type and dosage affect desensitization efficacy. Among the included studies, four types of milk interventions were used: baked milk, heated milk, raw cow milk, and glycated milk, with most studies using raw cow milk. Heating or baking milk at high temperatures can degrade certain structural epitopes, rendering them unrecognizable by allergen-specific IgE. Although one study (18) suggested that frequent consumption of baked products accelerates tolerance to cow milk protein, our study did not find this relationship, which is consistent with the meta-analysis results of Anagnostou et al. (41). Tolerance to heated or baked milk may be temporary and diminish within a few years. Dantzer et al. found that a protocol involving gradually increasing doses of baked milk was effective in promoting desensitization. This suggests that the initial dose may influence the efficacy of desensitization. Two studies evaluated the effects of low-dose and high-dose target protocols, and a pooled results indicated that the maximum dose did not appear to be associated with the efficacy of tolerance. Furthermore, baked or heated milk products are not standardized, and the extent and consistency of the allergen quantity or epitope structure in each preparation method are unknown, necessitating further research.

Although children with cow’s milk allergy have a higher prevalence of EoE than the general population, Ferreira Martins et al. (33) reported cases of children with CMA who had no pre-existing EoE developing EoE following oral immunotherapy. This suggests that EoE may represent a potential complication of OIT treatment. EoE typically emerges approximately 2.8 years after initiating the maintenance phase of OIT in CM-allergic patients (28). Therefore, studies with short follow-up periods may underestimate the true incidence of EoE. In our meta-analysis, the observation periods in the included RCTs ranged from 23 to 96 weeks, with only one study following patients for 96 weeks and the remainder not exceeding 52 weeks. This relatively short follow-up duration may have led to underestimation. After expanding our search to include non-RCT studies, we found that 4% of CM-allergic patients developed biopsy-confirmed EoE following OIT - a rate consistent with previous reports (42).

This study has several strengths: 1) inclusion of the latest research on immunotherapy and biological therapy for cow milk protein allergy, encompassing many participants, and 2) a comprehensive analysis with stratified evaluations of immunotherapy for CMA by region, age of inclusion, intervention methods, and control groups. However, the high heterogeneity across studies, including differences in study population, intervention protocols, outcome measures, and other methodological variations, presents a limitation. Nevertheless, the large sample size and the stability of sensitivity analysis results add robustness to our findings for clinical decision-making.

Limitations of this study included: 1) the included studies had a small sample size; 2) the exclusion of studies without a control group, potentially introducing bias in the evaluation of indicators before and after immunotherapy or biological therapy; 3) high heterogeneity among included studies and the failure to identify the sources of heterogeneity through subgroup analyses and other methods. The heterogeneity may be attributed to variations in target doses and maintenance duration across studies. The included studies in this analysis employed varying protocols, with target doses ranging from 3 mL to 250 mL. The high-dose group demonstrated a higher incidence of severe adverse reactions during the maintenance phase compared to the low-dose group. Longer durations of OIT were associated with higher rates of desensitization or non-responsiveness (43). Consequently, the treatment duration significantly impacts efficacy evaluation. The double-blind, placebo-controlled food challenge (DBPCFC) is the gold standard for diagnosing food allergies and is an objective tool for assessing desensitization therapy, allowing for the evaluation of changes from before to after immunotherapy and the assessment of thresholds. Notably, many articles did not use DBPCFC as an outcome measure, limiting the interpretation of the results. Inconsistent follow-up periods and potential publication bias further complicate the interpretation and generalization of the results. Optimizing the included population, standardizing trial design, and outcome measurements may help improve heterogeneity; 4) Sustained unresponsiveness is defined as the absence of allergic reactions upon food reintroduction after a prescribed period of allergen avoidance following OIT discontinuation, serving as a key metric for assessing long-term therapeutic success. However, none of the included RCTs reported sustained unresponsiveness rates, underscoring the need for future trials to incorporate this clinically meaningful endpoint;5) the lack of a systematic evaluation of long-term effects or side effects. The outcome measures included in this study had a relatively short duration, mostly within one year, and lacked long-term outcome evaluations, and 6) Among the included studies, only one assessed cytokine changes during OIT without in-depth analysis of the underlying immunomodulatory mechanisms. There are reports (44) that OIT may have long-term side effects, including eosinophilic esophagitis; however, most are case reports, and further attention is needed to monitor these side effects.

Future studies should establish standardized definitions for initial dosing (differentiating low versus standard starting doses), maintenance dosing (distinguishing low versus high maintenance doses), and efficacy evaluation criteria to reduce inter-study heterogeneity. A severity-stratified design should be implemented, with conservative low starting doses adopted for children with severe allergies. Furthermore, extended follow-up periods are recommended to properly evaluate long-term therapeutic efficacy and safety profiles.

## 5 Conclusion

This meta-analysis suggests that oral immunotherapy can effectively desensitize milk proteins in children with cow protein milk allergy, though data on long-term follow-up remains incomplete. Currently, there are limited data on the long-term use of immunotherapy agents as well as the impact of treatment interruption on milk allergy status, immunological changes, and improvements in quality of life. Although the reported adverse events are mainly mild to moderate, comprehensive studies on long-term safety are still lacking.

## Data Availability

The original contributions presented in the study are included in the article/**Supplementary Material**. Further inquiries can be directed to the corresponding author.
