# Supplementary material for: Oral immunotherapy for cow’s milk allergy in children: a systematic review and meta-analysis
Source: Front Immunol. 2025 Jun 4;16:1570050. doi: 10.3389/fimmu.2025.1570050 (PMC12175089; doi:10.3389/fimmu.2025.1570050)

**Supplementary data 1**

**Search strategy**

**Pubmed**

((((((Immunotherapy[MeSH Terms]) OR (baked Milk) OR (microwave-heated milk) OR (heated milk) AND ((((((((((((((Milk Hypersensitivity[MeSH Terms]) OR (Milk Hypersensitivities)) OR (Hypersensitivity, Milk)) OR (Milk Allergy)) OR (Allergy, Milk)) OR (Milk Allergies)) OR (Cow's Milk Protein Allergy)) OR (Cow's Milk Allergy)) OR (Allergy, Cow's Milk)) OR (Cow Milk Allergy)) OR (Cow's Milk Allergies)) OR (Cows Milk Allergy)) OR (Milk Allergy, Cow's)) OR (CMPA))

**Embase**

| No. | Query |
| --- | --- |
| #16 | #3 AND #15 |
| #15 | #6 OR #9 OR #14 |
| #14 | #10 OR #11 OR #13 |
| #13 | 'heated milk':ab,ti |
| #12 | 'microwave-heated milk':ab,ti |
| #11 | 'viaskin milk':ab,ti |
| #10 | 'baked milk'/exp |
| #9 | #7 OR #8 |
| #8 | 'fb 317':ab,ti OR 'fb317':ab,ti OR 'gbr 310':ab,ti OR 'gbr310':ab,ti OR 'hu 901':ab,ti OR 'hu901':ab,ti OR 'monoclonal antibody e 25':ab,ti OR 'monoclonal antibody e25':ab,ti OR 'olizumab':ab,ti OR 'rg 3648':ab,ti OR 'rg3648':ab,ti OR 'rhumab 25':ab,ti OR 'rhumab e25':ab,ti OR 'sti 004':ab,ti OR 'sti004':ab,ti OR 'syn 008':ab,ti OR 'syn008':ab,ti OR 'xolair':ab,ti OR 'omalizumab':ab,ti |
| #7 | 'omalizumab'/exp |
| #6 | #4 OR #5 |
| #5 | 'immune therapy':ab,ti OR 'immunogenic therapy':ab,ti OR 'immunoglobulin therapy':ab,ti OR 'immunological therapy':ab,ti OR 'immunological treatment':ab,ti OR 'immunomodulant therapy':ab,ti OR 'immunomodulary therapy':ab,ti OR 'immunomodulating therapy':ab,ti OR 'immunomodulation therapy':ab,ti OR 'immunomodulative therapy':ab,ti OR 'immunomodulator therapy':ab,ti OR 'immunomodulatory intervention':ab,ti OR 'immunomodulatory therapy':ab,ti OR 'immunomoduling therapy':ab,ti OR 'immunomodurating therapy':ab,ti OR 'immunotherapy':ab,ti |
| #4 | 'immunotherapy'/exp |
| #3 | #1 OR #2 |
| #2 | 'allergic reaction to cow`s milk'/exp OR 'allergic reaction to cow`s milk' OR 'allergic reaction to cows milk'/exp OR 'allergic reaction to cow’s milk' OR 'allergic reaction to milk'/exp OR 'allergic reaction to milk' OR 'allergic reactivity to cow milk'/exp OR 'allergic reactivity to cow milk' OR 'allergic to milk'/exp OR 'allergic to milk' OR 'allergy, cow milk'/exp OR 'allergy, cow milk' OR 'allergy, milk'/exp OR 'allergy, milk' OR 'cow milk allergy'/exp OR 'cow milk allergy' OR 'cow milk hypersensitivity'/exp OR 'cow milk hypersensitivity' OR 'hypersensitivity, cow milk'/exp OR 'hypersensitivity, cow milk' OR 'milk hypersensitivity'/exp OR 'milk hypersensitivity' OR 'milk protein allergy'/exp OR 'milk protein allergy' OR 'milk sensitivity'/exp OR 'milk sensitivity' OR 'serum milk antibody'/exp OR 'serum milk antibody' OR 'milk allergy':ab,ti |
| #1 | 'milk allergy'/exp |

**Cochrane**

| #1 | MeSH descriptor: [Milk Hypersensitivity] explode all trees | | |
| --- | --- | --- | --- |
| #2 | Cow's Milk Allergy | |  |
| #3 | cow's milk protein allergy | | |
| #4 | CMPA |  |  |
| #5 | #1 or #2 or #3 or #4 | |  |
| #6 | MeSH descriptor: [lmmunotherapy] explode all trees | | |
| #7 | MeSH descriptor: [Omalizumab] explode all trees | | |
| #8 | Viaskin Milk | |  |
| #9 | baked Milk | |  |
| #10 | microwave-heated milk | | |
| #11 | heated milk | |  |
| #12 | #6 or #7 or #8 or #9 or #10 or #11 | | |
| #13 | #5 and #12 | |  |

**Scopus**

(TITLE-ABS-KEY (immunotherapy) OR TITLE-ABS-KEY (baked AND milk) OR TITLE-ABS-KEY ( microwave-heated AND milk ) OR TITLE-ABS-KEY (heated AND milk) ) AND ( TITLE-ABS-KEY (milk AND hypersensitivity ) OR TITLE-ABS-KEY (cow's AND milk AND protein AND allergy) OR TITLE-ABS-KEY ( cow's AND milk AND allergy ) OR TITLE-ABS-KEY (cmpa) )

**sTable 1 Characteristics of included studies**

| **Author, year** | **Study design** | **Participants age** | **Location** | **Intervention** | **Comparator** | **Gender ratio（M:F）** | **Target dose (mg) of IT** | **Total treatment duration** | **Outcomes** |
| --- | --- | --- | --- | --- | --- | --- | --- | --- | --- |
| Boven,2023^11^ | RCT | 3m-36m | Dutch | Heated and glycated CM protein powder (iAGE product) | extensively hydrolysed formula | 6:5 (T)  6:1 (P) | 0.8-1.7g | 32 to 96 w | Outcome 1: passed DBPCFC (cumulative dosage 1444mg) Outcome 2: the changes in the following parameters were assessed at the final assessment |
| Yamamoto-Hanada, 2023^12^ | RCT | 1-18y | Japan | Combination of cow milk and Lactiplantibacillus plantarum YIT 0132 | Cow milk without probiotics | NA | 125 ml of LP0132 juice daily | 24 w | Outcome 1: threshold CM dose for triggering CMA increased in the final assessment CM challenge test  Outcome 2: the changes in the following parameters were assessed at the final assessment (1) baseline threshold CM dose; (2) serum levels of casein and β-lactoglobulin-specific immunoglobulin E (sIgE); (3) serum levels of casein and β-lactoglobulin-specific IgG4; and (4) the CM dose in CM-OIT. |
| Cohen,2022^9^ | RCT | 9-15y | Cananda | Cow milk | Avoidance | NA | 200 ml of milk (8 g of milk protein) | 52 w | Outcome: reach 200 ml milk consumption at 1y |
| Dantzer,2022^13^ | RCT | 3-18 y | USA | Baked cow milk | Placebo | 7:8(T)  9:6(P) | 2000 mg of baked milk protein daily | 52 w | outcome 1: tolerance of 4044 mg of cumulative baked CM protein (122.5 mL) at exit DBPCFC after 12 months of IT  Outcome 2: tolerance of 2044 mg of baked CM protein (61.9 mL) at exit challenge  Outcome 3: tolerance of 1044 mg of baked CM protein (31.6 mL) at exit challenge  Outcome 4: Change in maximum tolerated dose of baked milk within groups (baseline-exit)  Outcome 5: Change of quality life |
| Maeda, 2021^14^ | RCT | 3-12 y | Japan | Cow milk | Placebo | 7:7(T)  7:7(P) | 100ml CM daily | 52 w | Outcome 1: Tolerance to 100 ml cow's milk at challenge test  Outcome 2: the percent change in the threshold of cow's milk intake, result of skin prick test with cow's milk, eosinophil count, antigen-specific IgE levels, changes in antigen-specific IgG4 levels |
| Nagakura, 2021^15^ | RCT | 5-12y | Japan | Heated milk | Unheated milk | 14:3(HM)  11:5(UM) | 3 mL CM daily | 52 w | Outcome 1: the rate of total number of symptoms per total number of ingestions during the 12-month study.  Outcome 2: symptom severity, symptoms by organ  Outcome 3: the proportion of desensitization to 3 mL Outcome 4: passing the 3- and 25-mL OFC  Outcome 4: immunological change |
| Takaoka, 2020^16^ | RCT | 5-15y | Japan | High dose cow milk | Low dose cow milk | NA | the threshold with 20ml for low-dose group and 100ml for high- dose group | 26 w | Outcome 1: the threshold determined in the milk OFC after 6 months  Outcome 2: the number of symptoms and the number of epinephrine doses |
| Ogura, 2020^17^ | RCT | 3–15 y | Japan | High dose cow milk at maintenance phrase (100ml) | Low dose cow milk at maintenance phrase (25%-dose of 100ml) | 7:6 (100%CM)  11:2 (25%CM） | the threshold with 25ml for low-dose group and 100ml for high- dose group | 26 w  52 w | Outcomes 1: SU: the rate of no response to 3400 mg (100ml) milk in the first year (oral food challenge after 2-week ingestion cessation)  Outcomes 2: the rate of no response to 3400 mg milk in the second year  Outcomes 3: immunological changes  Outcomes 4: rates of allergic symptoms and medication used during OIT ingestion for 2 years |
| Esmaeilzadeh, 2018^18^ | RCT | 6m-3y | Iran | Baked milk | Avoid milk | 24:18(T)  27:15(C) | containing 4.6 g of milk protein | 52 w | Outcome 1: Passed the OFC with 240 ml of skim milk |
| Nowak-Wegrzyn,2017^19^ | RCT | 4 -10 y | USA | Baked milk products | Avoid milk | 95:41(T)23:11(Avoid) | consume baked milk products | 52 w | Outcome 1: tolerance to the non-baked milk  Outcome 2: Immunologic changes |
| Salmivesi,2016^27^ | RCT | 6–14 y | Finland | milk | Placebo | 8:10(T)  4:6(P) | 6400mg CM | 6m | Immunology markers in CMA and the changes in markers after OIT |
| Salmivesi,2013^20^ | RCT | 6–14 y | Finland | milk | Placebo | 8:10(T)  4:6(P) | 6400mg CM | 23w | Outcome 1: tolerate the 6400mg CM protein daily  Outcome 2: tolerate the 6400mg CM protein or equivalent amount of milk products daily after 6–12 months or 3-3.5y follow-up |
| Lee,2013^21^ | RCT | 7-12 m | Korea | milk | Avoid milk | 8:6(T) 5:7(Avoid) | 6600mg CM protein (200ml) daily | 6m | Outcome 1: tolerate 6600mg CM protein (200ml) daily after 6months  Outcome 2: the change of CM-specific IgE and CM-specific IgG4 |
| Martorell,2011^8^ | RCT | 2–3y | Spain | milk | Avoid milk | 19:11(T)  15:15(Avoid) | 6000mg CM protein (200ml) twice a day | 1y | Outcome 1: total tolerance (200 mL of cow’s milk);  Outcome 2: skin sensitivity to CM and threshold dose in DBPCFC, total IgE before desensitization, and specific IgE against milk and casein |
| Pajno,2010^22^ | RCT | 4-10y | Italy | milk | Soy milk | 8:7(T) 9:6(Soy milk) | 6600mg CM protein (200ml) daily | 4m | Outcome 1: tolerate 6600mg CM protein (200ml) daily after 4 months  Outcome 2: tolerate 3300mg (100 ml) at exit OFC after 4 months  Outcome 3: the change of CM-specific IgE and CM-specific IgG4 |
| Caminiti,2009^24^ | RCT | 5-10y | USA | milk | Soy milk | 6:4 (T)  2:1 (P) | 200ml CM daily | 4m | Outcome 1: tolerate 200ml CM daily after 4 months |
| Skripak,2008^25^ | RCT | 6-17 y | USA | Dry nonfat powdered milk | Placebo | 8:5(T)  4:3(P) | 500mg CM protein (15ml) daily | 23w | Outcome 1: the change of CTD during OFC atter 23w  Outcome 2: the change of CM-IgE and CM-IgG4 |
| Longo,2008^26^ | RCT | 5-17 y | Italy | milk | avoid | 21:9(T) 18:12 (avoid) | >150ml CM daily | 1y | Outcome 1: tolerate 150 mL of cow’s milk or more in a single dose  Outcome: partial tolerance: at least 5 mL but less than 150 mL in a single dose |
| Morisset, 2007^27^ | RCT | 13m-6.5y | France | Lactose-free milk | avoid | NA | 250ml CM daily | 6m | Outcome 1: tolerance to 200ml CM at SBPCFC at 6 months  Outcome 2: the change of SPT wheal size and specific IgE |

Abbreviations: DBPCFC: double- blind placebo-controlled food challenge; C: control; CM: cow's milk; CMA: cow's milk allergy; CRD: cumulative reactive dose; F: female; HM: heated milk; IT: immunotherapy; M: male; m: months; NA: not applicable; OFC: oral food challenge; OIT, oral immunotherapy; P: placebo; RCT: randomized clinical trial; SCD: successfully consumed dose; sIgE, specific Immunoglobulin E; SLIT: sublingual immunotherapy; SPT: skin prick test; SU, sustained unresponsiveness; T: treatment; UM: unheated milk; w: weeks; y: year;

**sFigure 1** Metanalysis of the rate of eosinophilic esophagitis after OIT


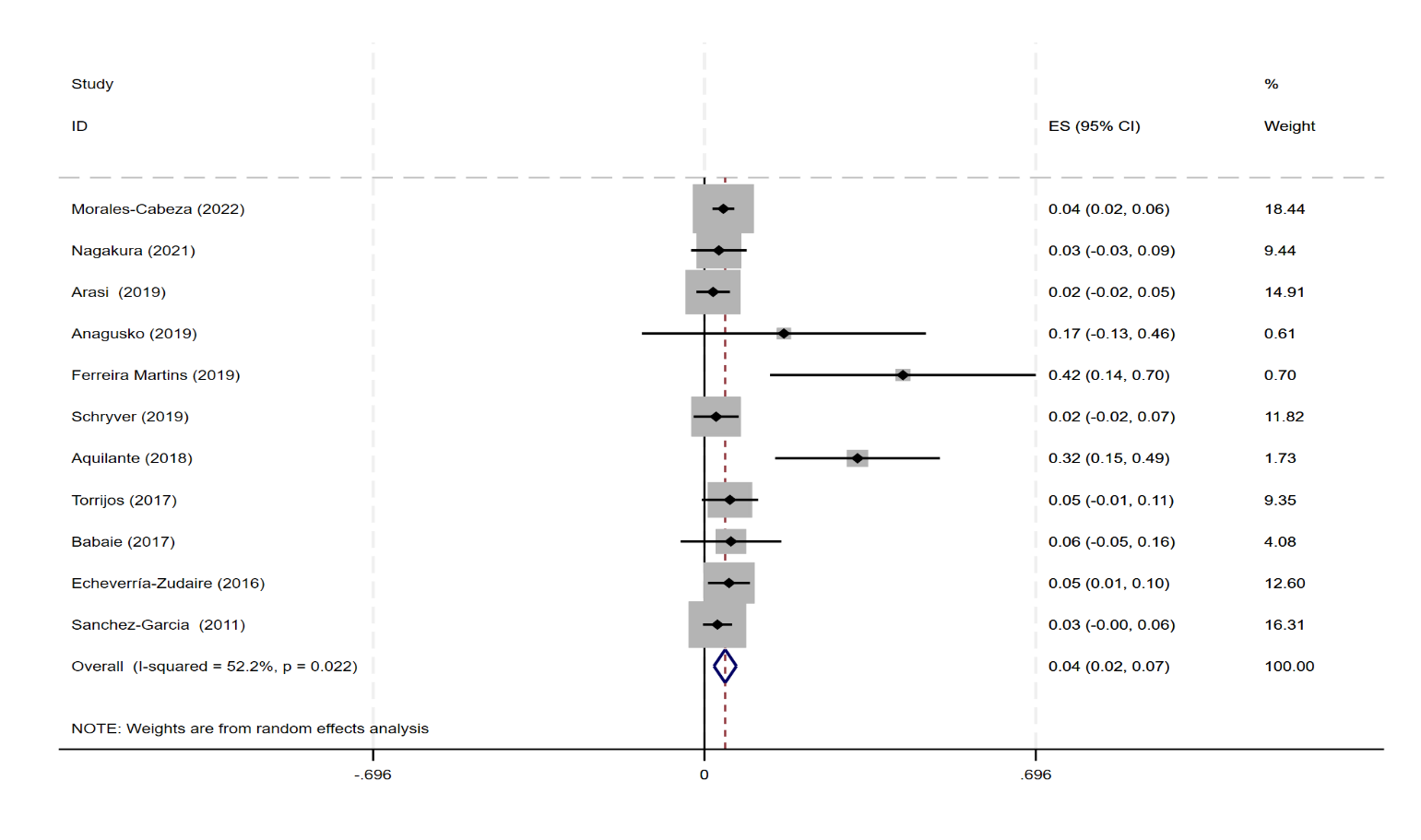


**sFigure 2** Change of cytokine levels induced by OIT in CMA


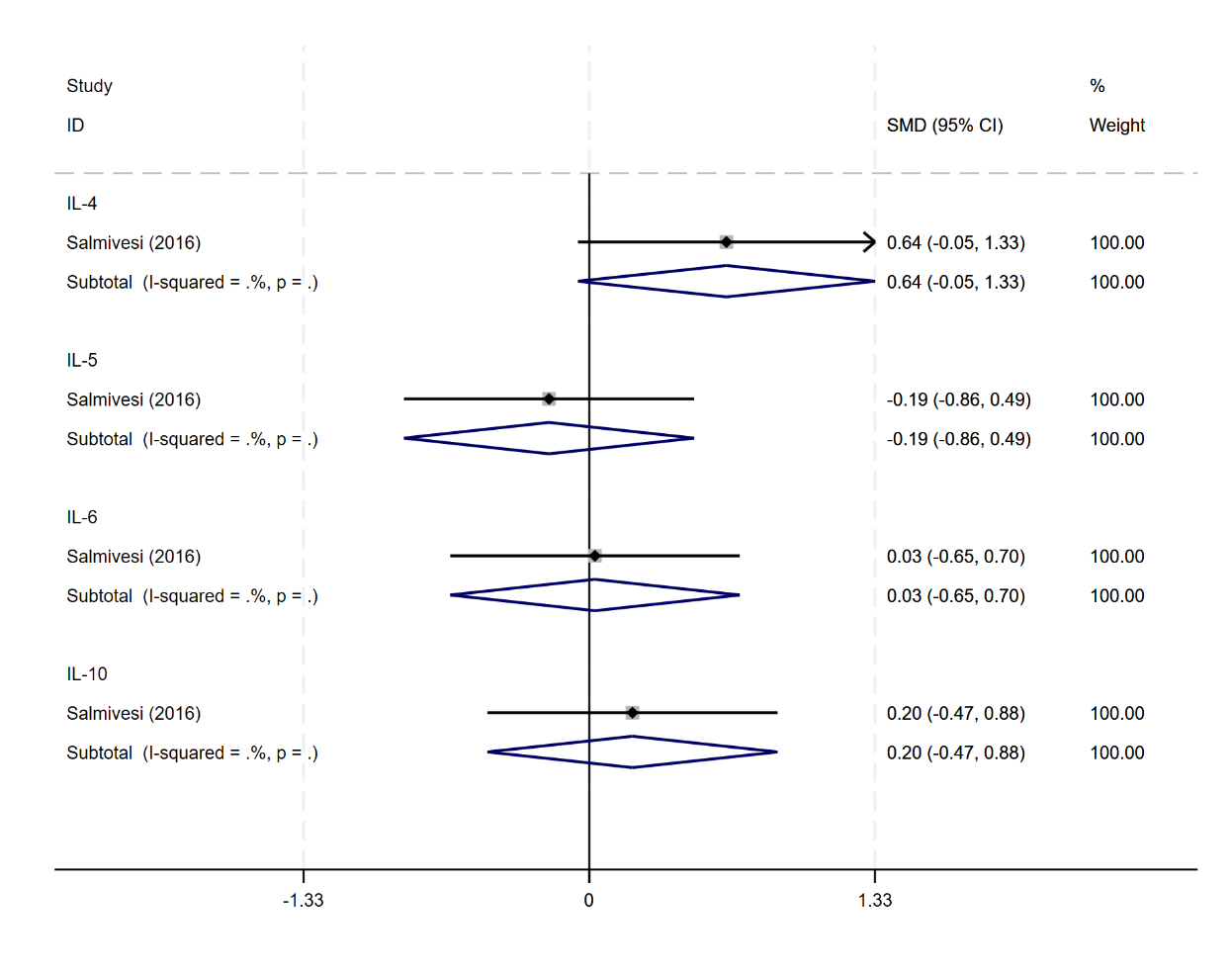


**sFigure 3** Sensitivity analysis of desensitization induced by OIT in CMA


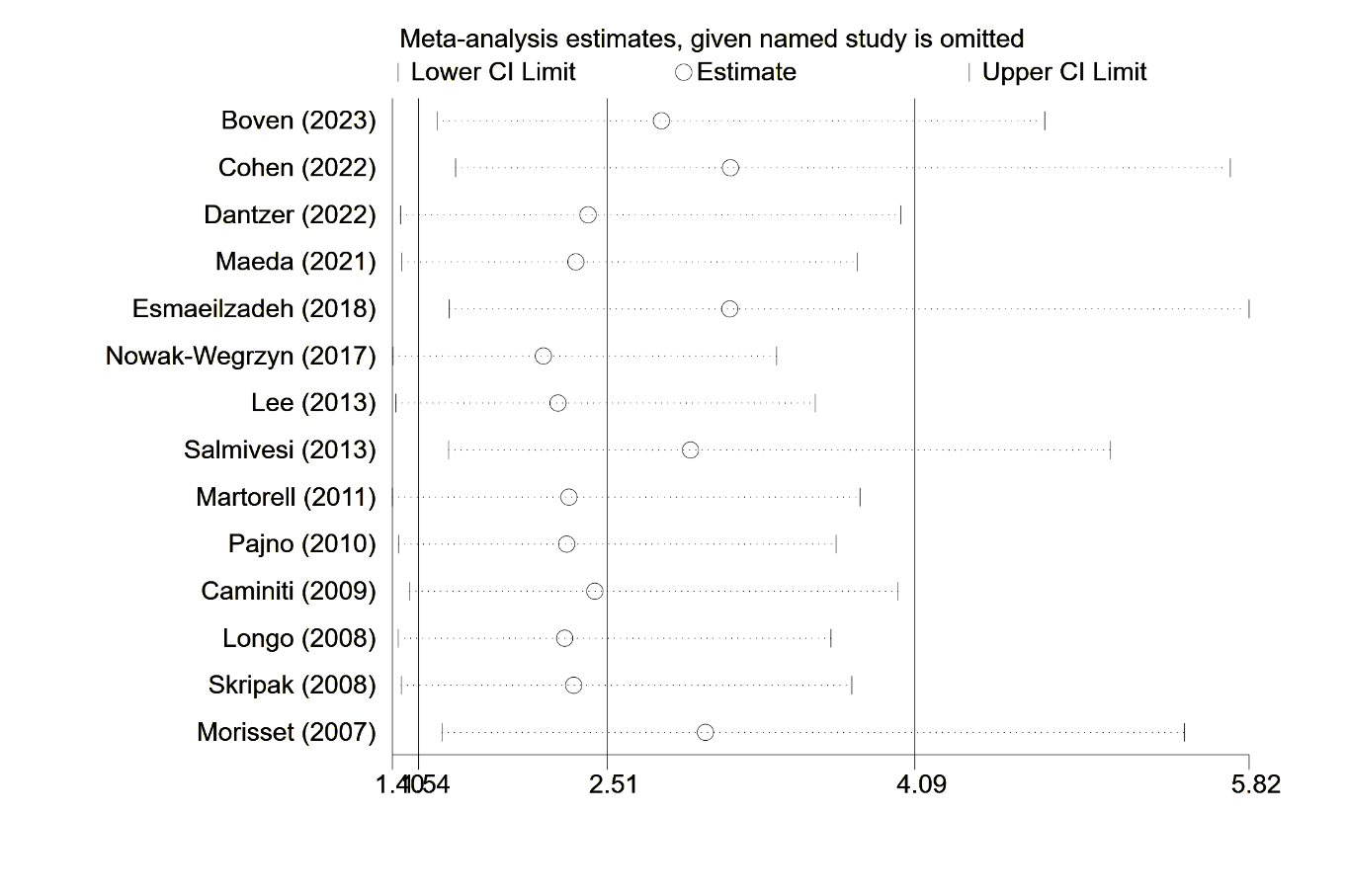


**sFigure 4** Sensitivity analysis of adverse events induced by OIT in CMA


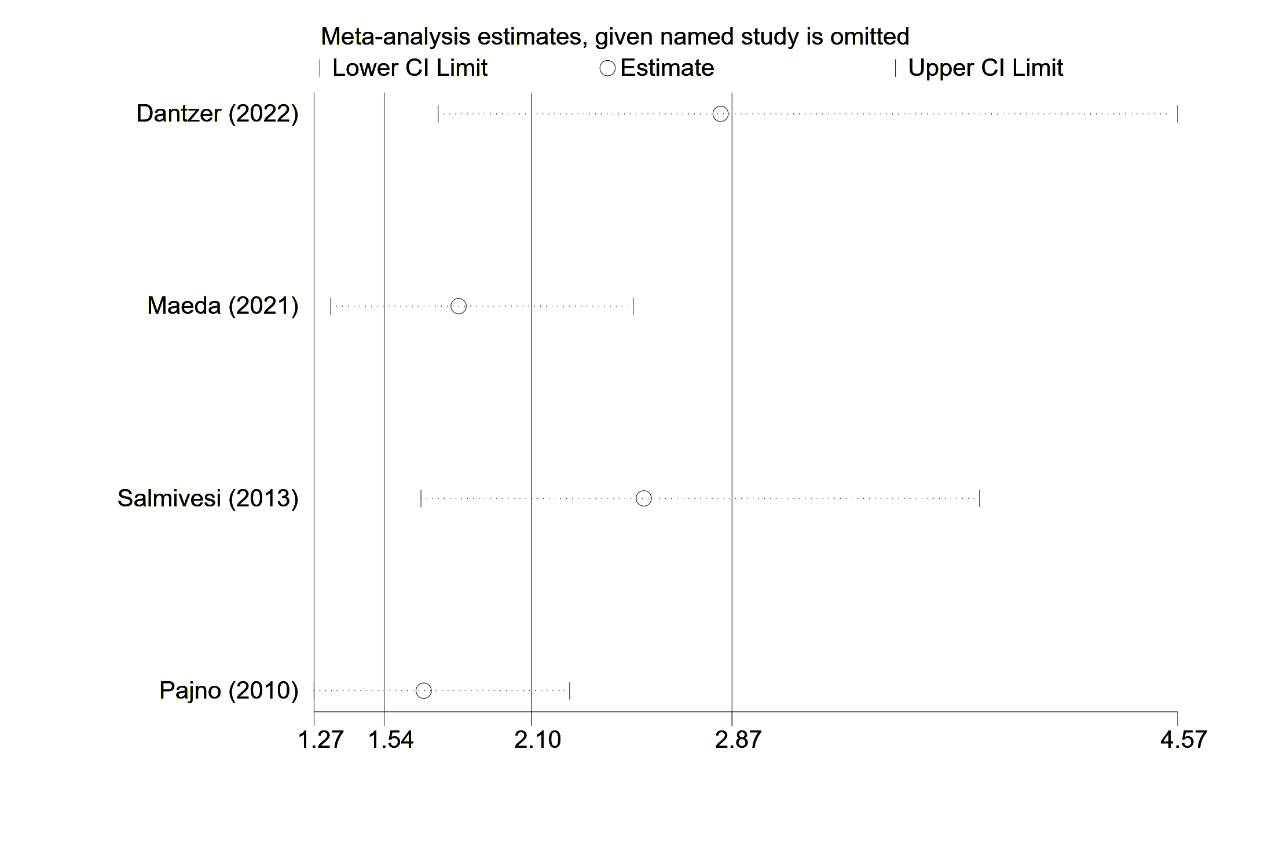


**sFigure 5** Publication bias and trim and fill method for desensitization induced by OIT in CMA

**(A)** Publication bias; (B) trim-and-fill method for publication bias


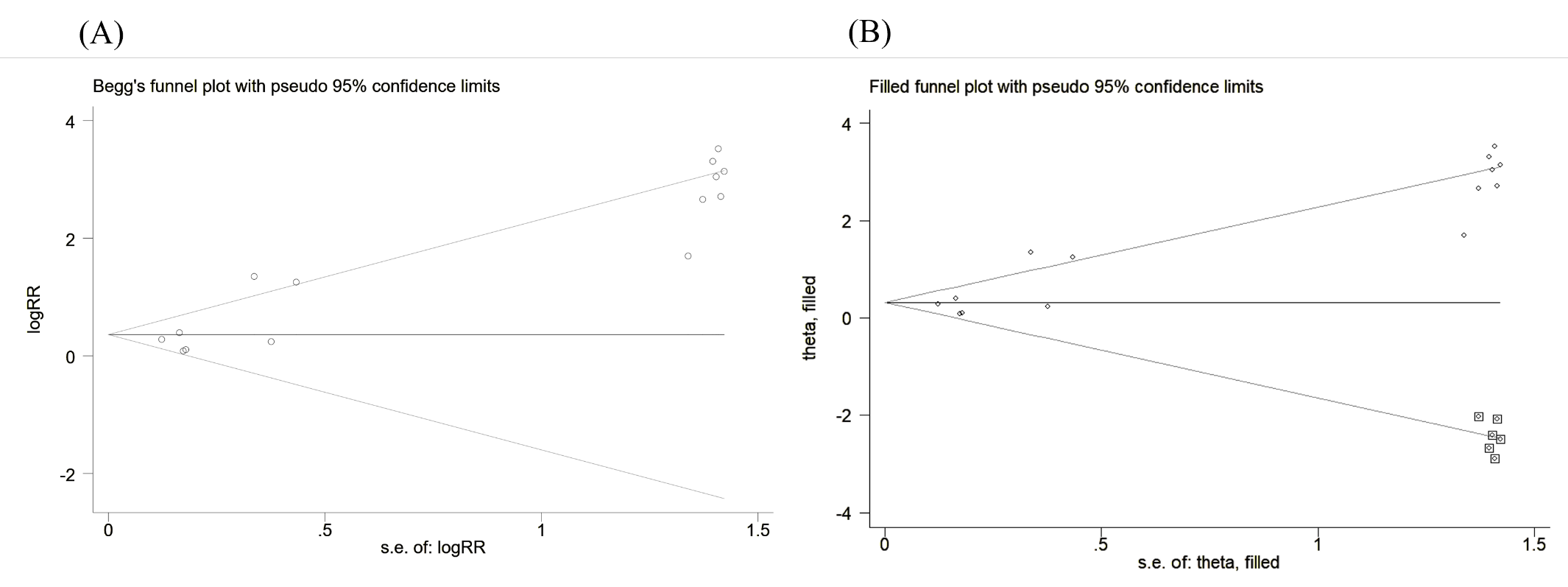

Supplement: Supplementary file 1 [file DataSheet1.docx]
